# Removable Restorations*An illustrated lecture given at the Golden Jubilee meeting of the Tennessee State Dental Association, Memphis, June, 1917. Reprinted from “The Dental Summary.”

**Published:** 1918-01

**Authors:** Dayton Dunbar Campbell

**Affiliations:** Kansas City, MO.


### REMOVABLE RESTORATIONS *

BY DAYTON DUNBAR CAMPBELL, D.D.S., KANSAS CITY, MO.

It is impossible to justify a dental restoration which does not have as its ultimate purpose the preservation or perfection of the normal occlusion of the teeth. Whatever may be said about causes of failure in dentistry, your essayist is firm in the belief that all of these occuring in cases of partial restoration point to faulty occlusion resulting in traumatic injury to the tissues enveloping the teeth, as the paramount cause. Faulty occlusion is due to the lack of an intimate working knowledge of the anatomical relationship of the tooth cusps in masticatory effort. This lack may be largely remedied through the use of ''study casts'' and the employment of devices, natural or artificial, permitting proper occlusion in the various stages of the progress of the work.

This paper, accordingly, seeks to emphasize the attainment of a perfect occlusion through the employment of partial restorations, and to consider in detail certain partial restorations which possess superior commendatory features.

The range of choice in restorations for an individual case makes the final selection of any one of these an act involving vital moral and ethical aspects. The larger the field of selection the more difficult the choice. We may narrow the field by keeping in mind the purpose underlying our endeavor to secure perfect occlusion, which is to preserve or improve the health and life of the patient. While emphasis is placed upon the *health* of the patient, we may incidentally remark that we have known some very healthful individuals who were poor specimens for the artist's purposes; we, therefore, note that a successful restor-

* An illustrated lecture given at the Golden Jubilee meeting of the Tennessee State Dental Association, Memphis, June, 1917. Reprinted from "The Dental Summary."

ation may contribute also to the esthetic appearance of the patient, besides enhancing his capacity to enjoy life and promoting his general efficiency.

It is scarcely necessary for the writer to suggest that under normal conditions the best service that can be rendered a patient is to preserve for him his natural teeth. It is no violation of this rule, however, to insist that a loss of all but two or three teeth in either arch justifies the elimination of these as well. In cases of pyorrhea it is very often the wisest method to advise that all of the teeth be extracted without delay, since a delay of two or three years may permit the resorption of the alveolar process to such a degree that nothing remains to withstand lateral stress. This is apparently the most difficult decision that the dentist is called upon to make and to maintain—that is, to say whether the remaining teeth with their small area of masticatory surface shall remain as aids in the retention of partial restorations, or be removed in order that complete restorations may be made. In the vast majority of cases the dentist is doubtful of his ability to provide something better, and the patient noting this, in the very manner of the dentist, is more firmly convinced regarding the negative virtues of anything that that dentist can make. The patient wins out in evident reliance upon the strength of the old saying that "a bird in the hand is worth two in the bush;" finally deciding that one tooth in the jaw is worth two on the plate. In the light of recent achievements by Dr. Rupert E. Hall, the above decision by both patient and dentist proves sadly erroneous.

With reference to cases warranting restorations which make use of the patient's own teeth in securing anchorage, the field of selection is immediately narrowed with practically unanimous consent by the positive and final rejection of the *large fixed* bridge. After this rejection, the field of possibilities ranges from the inexpensive, easily-constructed partial plate to the elaborate attachments and complicated retaining devices of removable bridge work—the inexpensive including clasps, Gilmore attachments, Roach attachments, the Cummer indirect retainer, while with the expensive may be enumerated the telescope crown, the split-pin, the split-bar, the Chayes attachments. In the latter class the dentist may, within certain acknowledged limits, give full play to the promptings of his genius. The writer does not question the superiority of the more expensive restorations to the less expensive, but he recognizes that the personal equation on the part of the patient determines the measure of superiority in comfort, in speech, in palatability of foods, and in ability to maintain a pride befitting one's station in life. There is no argument to support a dentist's recommendation of jeans and blue denim to a patient whose manner of living calls for broadcloth.

The primary source of satisfaction which a patient finds in wearing a minor restoration of the inexpensive kind may be more fully supplied through the more expensive devices. The truth is, that many minor restorations supplying one, two or more teeth are being worn with apparently full satisfaction to both patient and dentist—although the satisfaction is derived from a service which neither has primarily in mind. A concrete example of this is a small vulcanite denture replacing six-year molars in the lower jaw, which the patient wears with comfort and satisfaction—and the patient in reality masticates much better with than without it, but certainly not *upon* it. On second thought one would realize that the area sustaining the pressure of any force exerted upon the denture equal to that exerted upon the adjacent teeth would be accompanied by pain and discomfort. The service rendered is not in the supposedly increased masticatory surface provided but in the ease with which, now, the bolus of food in its masticatory excursions is kept on the masticatory surfaces of the teeth. The food, in its excursions from lingual to buccal positions, does not have to be rescued each time from the otherwise edentulous space. Your writer is constrained to believe that this feature of partial plates commends them unconsciously to the use of the wearer, more than any other feature save perhaps the esthetic and cosmetic features. I have no record in my experience of a case in which either a partial upper or lower or both are being worn, but that the real incisive or triturating process is being carried forward on the remaining natural teeth. If this condition does not prevail when first inserted it results within a few weeks, as a consequence of recession, resorption or other causes.

The superiority of the superior grade of workmanship is best presented through the use of study casts, the practical aid of which for many purposes can not be over-estimated. During the past few years it has been the writer's pleasure to meet a great many dentists in different parts of the country, and of the entire number there have been few who ever gave any indication of having previously visualized the completed case before the work was begun. How many of us have a mental picture of the occlusion which our proposed restoration will furnish? There is not a shadow of a doubt in my mind that time spent on the study of occlusion is a hundred-fold more profitable than the same amount of time devoted to the invention of intricate attachments or to the elaboration of patent devices. No dentist should feel any embarrassment in telling his patient that he is unable to say without first studying the case just what he will recommend. Nothing is so well calculated to inspire your patient with confidence in your ability to meet the exigencies of that particular case than is a submission of a number of devices or methods and their comparative advantages and disadvantages—including the economic or financial considerations also. The contemplated piece may be constructed from soft copper wire and wax, and painted with gold bronze. Casts are as serviceable to the patient in determining a selection as tailor-made suits are superior to samples of cloth from a mail-order house. The construction is simple and whether you call it salesmanship or "the graphic portrayal of the possibilities of the case," the results are the same. The ease and simplicity with which an impression in modeling compound is taken for a study cast offers no excuse for careless impressions, although it is too often the explanation for them.

The most successful construction of restorations where the occlusion is to be determined outside of the mouth is achieved through the use of the Hall articulator. Occlusion may be defined as that intimate relation of the tooth cusps that provides for milling, grinding, triturating contacts which thoroughly disintegrate the morsel of food while the force employed is directed parallel with the long axis of the tooth. This involves the relationship of the tooth to its socket and to the tooth with which it is in antagonization. In this connection the writer believes that he is the first to suggest the use of the Hall articulator in the construction of bridge work—a suggestion made a year ago while a guest of the Nebraska state meeting. The principle upon which the Hall articulator is built is diametrically opposed to that of all others on the market, in that it takes no cognizance of the movement of the condyle in the glenoid fossa but instead depends upon the tactile sense of the tooth cusp. However, the angle of the tooth cusp is in full dentures arbitrarily fixed.

In partial restorations full casts of the upper and lower jaws preserving intact the remaining teeth, with the antagonizing cusps in synchronous or harmonious cusp relationship, are mounted on the Hall articulator in order to provide the normal individual movement which is to be incorporated in the partial denture under construction. The edentulous portion of the case is supplied with the artificial substitutes and each one ground to correspond with the angle already established by the natural teeth. The grinding is continued on the basis of the direction pointed out by lateral jaw movements as determined by the tooth cusps. Since the bite is no uncertain factor in partial restorations, there being landmarks sufficient, a try-in is, so far as occlusion is concerned, a waste of time, and furthermore is not in the majority of cases accompanied by satisfactory results. To state briefly the substance of this effort, we may say: Aim to secure for each tooth, natural or artificial, an individual occlusion.

Where the restoration is to be of teeth attached with vulcanite, the bite is not opened on the articulator, since the teeth, though every precaution has been taken in packing and closing the flask, will come from the vulcanizer protruding from the vulcanite sufficiently to allow material for the final grinding of the occlusion in the mouth. In making corrections with the aid of artificial restorations the dentist ordinarily neglects to avail himself of the aid which liberties with the natural teeth afford. Especially is this neglect manifest with reference to teeth which during a long period have been of no service in mastication; for example, long cusps that have not been used for years should be cut off to approximate those which have been in actual service continually. It is wise to apprise the patient of the fact that decay can not occur upon these ground surfaces if they are continually used in mastication. This method of perfecting the occlusion is, of course, not new, but it is so indispensable to success that it needs to be emphasized. As much as possible of No. 120 carborundum powder, or No. 100 grit, is mixed with tooth paste and placed upon the surfaces of the teeth when the patient is requested to chew. The flow of saliva is very quickly so profuse that the grinding material is washed away. Oft-times from six to eight applications and from fifteen to twenty minutes are required. While this would be a very disagreeable process for individuals with all their natural teeth, the grinding material confined to the artificial cusps provides no unpleasant sensations such as the patient anticipates.

What proportion of dentists insist on supplying porcelain teeth in the case of bicuspid and molar restorations, when they know full well that the porcelain tooth cusp provides a masticatory surface incomparably superior to the gold crown from which the food slides like soap from a marble stand? In this one respect, a comparatively poorly-constructed and illy-adapted partial plate is superior to the finest piece of stationary bridge work with gold cusps and inaccessible areas in which particles of decaying food collect.

No complete set of instructions for the taking of impressions may be given, because the variations of a method are as numerous as the possible changes which may be evolved in a series of thirty-two units—which of course is almost beyond computation. As a general rule, the more difficult the case the greater the need for taking a plaster impression. For those cases in which posterior teeth are remaining, orthodontia trays are indicated. (This statement will fall as a hard blow to many who would like to find an easy way, but after the technic is mastered there is no easier way.) Where there are two or more intervening spaces to be supplied, the articulated casts are mounted on the articulator and the porcelain teeth ground and set up to close approximal contact with the adjacent plaster teeth; the case is then waxed up and the usual procedure of vulcanizing carried to completion. The case is finished to the sand papering and carried to the mouth and back to the lathe repeatedly and cut freely until the intricacies of its insertion are mastered. The teeth are now removed from the vulcanite by heating over a small alcohol flame, the holes left in the vulcanite by the pins are enlarged with a large bur and the teeth reset with hard wax. That portion of the plate conforming to the tissues is now cut away and modeling compound in stick form is applied with dry heat, and a removable impression is taken of the ridge and adjacent teeth. The case is now waxed up and invested, the flask opened and everything except the teeth taken out and thrown away. New rubber is vulcanized and when the case is finished it goes to place as did the plate impression.

For a case in which there are six or eight of the anterior teeth standing, the method of taking the impression is very similar to that used in taking full upper or lower impressions. Take for example a case where the patient has in the upper jaw six anterior teeth and the first bicuspids. To provide an efficient restoration for this case, the method of procedure, simplified as much as possible, is as follows: Select an over-size tray similar to that used for a full upper impression and fill with Hall's Black Impression Tray Compound. The impression is taken without endeavoring to secure detail on the labial and buccal borders of the teeth. Removing this impression from the mouth it is chilled and then removed from the tray. To meet the requirements of an almost perfect tray the impression is trimmed to meet the needs of the individual case, being thinned down to the cervical margins of the teeth, since there is no attempt to obtain an impression of these. The Hall technic for full impressions is then followed in detail. This is, in substance, as follows: Hot water, potassium sulphate and cast plaster are mixed very thin and stirred constantly until setting begins, when it is introduced into the fashioned tray and settled into position. Dr. M. M. House, of Indianapolis, in this connection found that cast plaster used, together with potassium sulphate, sets under saliva and slowly enough to allow time for careful manipulation. After it has set, the impression is removed from the mouth, and after drying for fifteen or twenty minutes is coated with an alcoholic solution of shellac which serves as a filler; when this is dry it is further coated with an alcoholic solution of sandarac to give a smooth surface. The casts are now poured with Stewart J. Spence's plaster compound—in such a way as to obviate the necessity of trimming the flint-like casts—the impression having been boxed up previously with Harbutt's plasticine (kindergarten modeling material), in order to provide a convenient matrix. Upon the cast a bite plate is constructed of Graft's base-plate compound and hard pink wax. This is taken to the mouth and the bite secured, and at this sitting an impression of the lower is taken in modeling compound. This cast from the lower is placed in position on the bite plate and mounted on the articulator. Just before the teeth are placed in position, a piece of Ney's oro elastic gold for each side is flattened at one end and bent at right angles and heated, then incorporated in the Graft's base plate, just anterior to the first tooth on the plate, the free ends extending in a buccal direction about a half inch. Another piece is extended along the side of the cast in the direction of the central incisor. This piece is called by Dr. W. E. Cummer, of Toronto, an "indirect retainer." It serves as a lever to prevent the plate from dropping down into the mouth. (How it serves this purpose will be shown in the clinic.) The teeth are now set up to proper occlusion and the case vulcanized. When finished and polished it is taken to the mouth where, with a round-nose plier, the wire is bent to conform to the neck of the tooth.

Where the clasp is not to be rigid, the writer prefers a wire one, since subsequent adjustments, when necessitated as a result of settling, may be more easily made. The writer further observes that in the clasp formed with pliers or peened out of sheet clasp gold, there is really but one line of serviceable contact about the circumference of the tooth, no matter what the width of the band. In other words, the open-end, hand-made clasp does not fit like a ferrule on a cane—and this is no reflection upon skilled workmanship; the securing of such adaptation is simply a physical impossibility. The wide clasp has the additional disadvantage of harboring the products of fermentation, which constitute the one factor known to be responsible for the disintegration of the tooth substance. It is a common belief among dentists that to clasp a tooth means to destroy that tooth through abrasion within a few years' time—another erroneous belief. The fact that a natural cusp when in antagonization with a platinized hard gold inlay does not suffer, but on the contrary makes marked impression upon the surface of the inlay, supports our observation that a belief in the destruction of teeth through clasp abrasion is unfounded. It may be relevant at this point for the writer to suggest that he prefers and teaches in the construction of form-fitting clasps, the employment of a method which does not suffer through an individual's faulty finger technic. This preferred method avails itself of the laws of physics, and is none other than the one utilized in casting gold inlays. Further specific reference to this method is embodied in the following presentation of the "grasshopper-leg adjunct:"

The grasshopper-leg adjunct is not itself an attachment, but it is an adjunct to the snug-fitting clasp or removable attachment such as the split-pin or split-bar types. Credit for the invention of this device is difficult to assign, but your essayist has been employing it with varying degrees of success, during the past five or six years. During this period the highest grade of clasp gold on the market has been used in its construction, but the limit of elasticity of all grades was soon reached and the molecular tension developed under constant use caused the leg to break at the knee. This difficulty has been eliminated since the introduction of elastic gold according to Louis J. Weinstein's formula. This gold is found to possess the "spring" quality necessary to assure the grasshopper-leg adjunct a permanent place in prosthetic dentistry and win for it increasing favor in connection with minor removable restorations.

The function of this device is not to compensate for settling, but is rather a provision for the resiliency of the tissue in the area underlying the base. The stress of mastication is distributed between the spring and the mucosa, the stress falling first upon the spring, which, yielding, allows the stress to be exerted finally against the mucosa itself; as soon as the stress is removed the spring is released from its state of tension and the mucosa then returns to normal. Thus, this adjunct allows the mucosa to remain normal and therefore requires in the clasp or other device no subsequent alteration such as is required when the tissues permanently recede. Another advantage almost too obvious to mention attends the use of this adjunct, namely, the absence of harmful leverage on the abutment tooth, since we are enabled to direct the force of mastication along a line more nearly parallel with the long axis of the tooth.

The construction of clasp and leg is, briefly, as follows: An inlay-investment reproduction of the tooth to be clasped is waxed up to include two opposing convex surfaces and an occlusal rest. The tooth and wax pattern are then invested and the usual inlay procedure is followed, except that a twelve per cent. platinum-alloyed gold is used, which properly may be designated as clasp metal. The secret of success in casting clasps lies in casting them at the lowest temperature possible, since incandescence permits a destruction of the balanced relationship among the elements of the clasp metal.

The leg in constructed in most instances of 16-gauge Ney's oro elastic gold wire. After bending the wire at right angles it is annealed and further bent back upon itself, and the whole adapted to meet the needs of the individual case, one end being soldered to the clasp and the other end left free until assembled in the mouth. The base to receive the clasp is waxed up with hard pink baseplate wax, the procedure being the same as that followed preparatory to taking the bite, except that a space about ten millimeters in width is left between the wax and the abutment tooth, that is, the tooth which is to receive the clasp. The clasp is placed in position on the tooth and the base is inserted; the patient is requested to close and the base is settled into place; plaster is now inserted between the abutment tooth and the base, and this relation maintained until the plaster is hardened sufficiently to be removed. The whole is invested in bridge investment material and the lower portion of the leg soldered to the lingual bar of the gold base—or in the event of a vulcanite base, the lower portion of the leg is extended back into the rubber sufficiently to insure stability when vulcanized.

The fact that the grasshopper-leg adjunct may be used with any unyielding appliance in order to assist in preserving resiliency is, perhaps, its highest recommendation.

